# Fatigue and Mental Illness Symptoms in Long COVID: Protocol for a Prospective Cohort Multicenter Observational Study

**DOI:** 10.2196/51820

**Published:** 2024-01-19

**Authors:** Ligia Pires, Cláudia Reis, Ana Rita Mesquita Facão, Armin Moniri, Ana Marreiros, Marta Drummond, Joana Berger-Estilita

**Affiliations:** 1 Serviço de Pneumologia Hospital de Portimão Centro Hospitalar Universitário do Algarve Portimão Portugal; 2 Serviço de Psiquiatria Hospital de Portimão Centro Hospitalar Universitário do Algarve Portimão Portugal; 3 Department of Otorhinolaryngology Sahlgrenska University Hospital University of Gothenburg Gothenburg Sweden; 4 Regenerative Medicine Program Department of Biomedical Sciences and Medicine University of Algarve Faro Portugal; 5 Algarve Biomedical Center University of Algarve Faro Portugal; 6 Hospital Particular do Algarve Faro Portugal; 7 Centro de Responsabilidade Integrada Sono e VNI Faculdade de Medicina Universidade do Porto Porto Portugal; 8 Institute of Anaesthesiology and Intensive Care Salem Spital Hirslanden Medical Group Bern Switzerland; 9 Institute for Medical Education University of Bern Bern Switzerland; 10 Centre for Health Technology and Services Research (CINTESIS) Faculty of Medicine Universidade do Porto Porto Portugal

**Keywords:** SARS-CoV-2, coronavirus, COVID, long COVID, fatigue, tired, tiredness, anxiety, depression, PTSD, stress, quality of life, mental health, post-COVID-condition, neuropsychological, neuropsychology, psychological, long COVID-19, COVID-19, myalgia, correlation, impairment

## Abstract

**Background:**

The aftermath of the COVID-19 pandemic continues to affect millions worldwide, resulting in persisting postvirus complaints and impacting peoples’ quality of life. Long COVID, characterized by lingering symptoms like fatigue and mental illness, can extend beyond a few months, necessitating further research to understand its implications.

**Objective:**

This study aims to quantify the degree of physical and psychological fatigue in patients following COVID-19 infection and examine its correlation with mental health disorders.

**Methods:**

Using a consecutive nonrandom sampling technique, we will conduct a prospective cohort multicenter observational study in 5 Portuguese hospitals. Symptomatic adult patients with previous COVID-19 attending follow-up consultations will be enrolled. We will include patients who had mild, moderate, and severe acute disease. We will assess clinical outcomes related to COVID-19, including the type of respiratory support such as high-flow nasal cannula, noninvasive ventilation, and invasive mechanical ventilation. The exclusion criteria will include previous severe psychiatric disorders confirmed by a psychiatrist; refusal or inability to respond to the questionnaire; concomitant neurological disorder; persistent fatigue symptoms during the 6 months before infection; and the need for invasive mechanical ventilation during COVID-19 infection due to a high prevalence of postintensive care syndrome. Our primary outcome is the prevalence of fatigue in patients with post–COVID-19 depression and/or anxiety, as measured by the Chalder Fatigue Scale (CFQ-11) and the Hospital Anxiety and Depression Scale (HADS). The secondary outcomes will include an assessment of health-related quality of life via the EQ-5D questionnaire and an exploration of the prevalence of symptoms of posttraumatic stress disorder (PTSD) using the 14-item Posttraumatic Stress Scale (PTSS-14). We will also examine the association between mental health symptoms and the severity of acute COVID-19. The post–COVID-19 data will be collected at least 6 months after the positive test and no longer than 9 months during the clinical appointment.

**Results:**

We expect our multicenter study on patients post COVID-19 to reveal a significant link between mental illness symptoms and both physical and psychological fatigue. Patients with heightened depression and anxiety may report increased levels of fatigue. Additionally, we expect to find persistent PTSD symptoms in a subset of participants, indicating the enduring psychological impact of the virus.

**Conclusions:**

This study may underscore the need for integrated care addressing physical and mental health in patients post COVID-19. The observed connections emphasize the importance of considering mental well-being for long-term health outcomes. Despite study limitations, our findings contribute valuable insights for future treatment strategies and highlight the necessity for comprehensive mental health support in post–COVID-19 care. This research provides valuable insights into the mental health implications of COVID-19 and its impact on post–COVID-19 fatigue and the overall well-being of affected individuals.

**Trial Registration:**

ClinicalTrials.gov NCT05323318; https://clinicaltrials.gov/study/NCT05323318

**International Registered Report Identifier (IRRID):**

DERR1-10.2196/51820

## Introduction

Despite the World Health Organization (WHO) declaration marking the end of the COVID-19 pandemic on May 5, 2023 [1], millions of people continue to have postvirus complaints. Moreover, the virus remains endemic in many parts of the world. Confirmed COVID-19 cases have exceeded 430 million globally, with 200 million in Europe alone [2]. The causative agent of COVID-19 is the novel SARS-CoV-2 [3]. Although, as the name implies, respiratory symptoms are acute, the term long COVID (or post-COVID syndrome or long-haul COVID-19) began gaining recognition in the scientific and medical communities after the first descriptions of long-lasting symptoms related to mental health, such as anxiety or stress after the first infection [4]. While the definition of long COVID is unclear, the most frequent symptoms are fatigue and dyspnea [5,6]. Other less typical symptoms include cognitive and mental disorders, headache, myalgia, chest and joint pain, smell and taste dysfunction, cough, hair loss, insomnia, wheezing, rhinorrhea, sputum, and cardiac and gastrointestinal issues [4]. These symptoms may persist for up to 6 months after hospital discharge, severely impacting patients’ quality of life [7]. Since July 2021, long COVID is considered a disability under the *Americans With Disabilities* Act [8].

Per its definition, long COVID appears within 3 months after the onset of COVID-19, with symptoms lasting for at least 2 months that an alternative diagnosis [9] cannot explain, including, myalgic encephalomyelitis and chronic fatigue syndrome. However, studies have reported different persistent symptoms in contrasting durations and frequencies among survivors of long COVID [10,11]. Long COVID appears like other postviral syndromes observed in other coronavirus diseases. For example, symptoms of fatigue, myalgia, and psychiatric impairments have affected survivors of Middle East respiratory syndrome (MERS) and those with severe acute respiratory syndrome (SARS) for up to 4 years [10,12,13]. Even at 7-year and 15-year follow-ups, pulmonary and bone radiological complications were evident among a proportion of survivors of SARS who were predominantly younger than 40 years [10,14,15]. This is unsettling, as it implies that long COVID may extend beyond just a few months.

Fatigue, a primary persistent symptom of long COVID, has been reported in 10% to 70% of patients [16-19]. It is defined as “a decrease in physical or mental performance that results from changes in central, psychological, or peripheral factors due to the COVID-19 disease” [20]. Thus, post–COVID-19 fatigue depends on conditional and psychophysiological factors comprising the individual’s task, environment, physical, and mental capacity, as well as the disease’s central, psychological, and peripheral aspects [20].

While fatigue is not traditionally considered a neuropsychiatric disorder, it can be a symptom of many mental health conditions, such as depression, anxiety, and posttraumatic stress disorder (PTSD) [21,22]. Therefore, it is often included in discussions of mental health symptoms in post–COVID-19 syndrome [23]. The term “neuropsychiatric” often refers to a wide range of disorders affecting the brain and mental health. In contrast, “neuropsychological” refers to studying the relationship between brain function and behavior [24]. The primary focus of this study is on psychiatric symptoms, particularly depression and anxiety. However, we will also investigate PTSD, which is a neuropsychiatric disorder involving both neurological and psychiatric aspects. In addition, the health-related quality of life (HRQoL) assessment includes a section that evaluates aspects related to anxiety and depression. While not designed for diagnosing psychiatric disorders, this assessment can help gauge how these symptoms affect a person’s overall quality of life, making it more of a general health assessment.

The current literature on long COVID and its mental sequelae has relied on self-reported symptoms through questionnaires administered either in-person or through telephone interviews to comply with public health guidelines. However, the correlation between these self-reported symptoms needs to be clarified. There is an obvious need to accurately characterize the mental sequelae of COVID-19 and the risk factors associated with these outcomes.

This study aims to quantify the degree of physical and psychological fatigue in patients post COVID-19 and assess the correlation of fatigue with other mental sequelae—particularly depression, anxiety, and PTSD. Furthermore, we aim to explore its impact on HRQoL.

Our research questions for this study are outlined in Textbox 1.

**Research questions of the study.**

Research Question 1 (primary outcome):

- Do patients with long COVID who experience depression and/or anxiety symptoms have a higher prevalence of fatigue?

Research questions 2 to 4 (secondary outcomes):

- Is there a correlation between the type of fatigue and depression and/or anxiety symptoms among patients post COVID-19?
- Is there a correlation between fatigue and the presence of PTSD symptoms in patients post COVID-19?
- Do patients with fatigue associated with mental health disorders have a lower health-related quality of life (HRQoL) after COVID-19?

## Methods

### Overview

This protocol adheres to the SPIRIT (Standard Protocol Items: Recommendations for Interventional Trials) statement [25]. This research is designed as an observational prospective cohort study.

### Recruitment

Our study will be conducted in 1 private and 4 public Portuguese hospitals that have established follow-up post–COVID-19 medical consultations. Patients who meet the eligibility criteria will be invited to participate in the study at the end of their appointments in the follow-up clinics. This study will be conducted using a consecutive, nonrandom sampling technique.

### Inclusion Criteria

Patients who cumulatively meet the following criteria will be eligible for inclusion: (1) ≥18 years; (2) previous case of COVID-19 at least 6 months after the diagnosis that is duly documented in the clinical record; (3) persistent symptoms after COVID-19 recovery, as defined by the WHO [9]; and (4) SARS-CoV-2 RNA confirmed by a positive real-time reverse-transcription polymerase chain reaction (RT-PCR) on a nasopharyngeal swab or SARS-CoV-2 antigen confirmed with a nasopharyngeal swab by a health care professional within 7 days of initial symptoms.

We will implement multiple measures to exclude the possibility of second infections and ensure that the individuals included have persistent symptoms following their initial COVID-19 recovery. Our inclusion criteria stipulate that individuals must have a positive test result for SARS-CoV-2 RNA or antigen within 7 days after their initial infection. Additionally, we will conduct thorough reviews of medical records and clinical assessments to assess symptom presentation and timing, differentiating between persistent cases and new infections. In cases of uncertainty or potential overlap with new infections, we are prepared to conduct repeat testing, which may include RT-PCR and genomic sequencing for accurate classification.

### Exclusion Criteria

Patients who meet the following criteria will be excluded from participation: (1) preexisting psychiatric disorders diagnosed by a psychiatrist before contracting COVID-19; (2) inability to respond to the questionnaire; (3) concurrent neurological disorders, such as stroke with sequelae, Alzheimer disease, and Parkinson disease; (4) individuals subjected to invasive mechanical ventilation during their COVID-19 infection; or (5) those experiencing persistent fatigue symptoms within the 6 months before SARS-CoV 2 infection.

**Figure 1:**
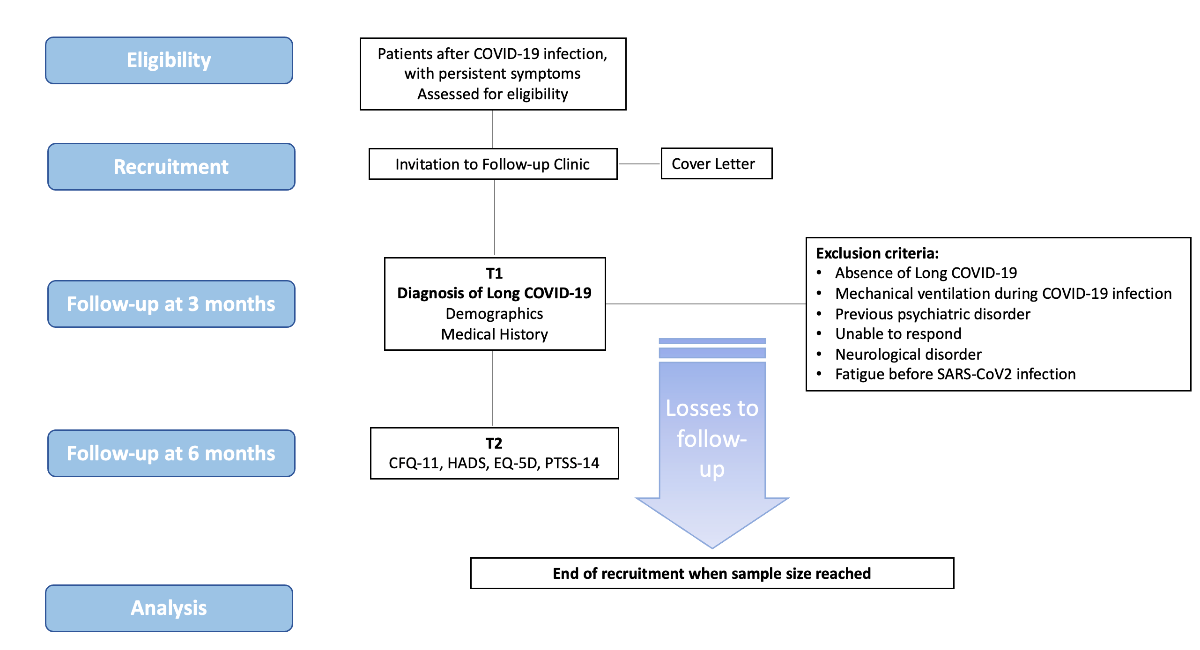
Study flowchart. CFQ-11: Chalder Fatigue Scale; HADS: Hospital Anxiety and Depression Scale; PTSS-14: 14-item Posttraumatic Stress Scale.

The study design and inclusion criteria will be reviewed regularly to ensure they align with current best practices and the latest understanding of COVID-19. A patient enrollment flowchart is presented in Figure 1.

### Data Collection

Data collection will take place during appointments at the long COVID follow-up clinics in all participating hospitals. Qualified clinical study staff will gather the data using a clinical research form (CRF) (Multimedia Appendix 1). These individuals comprise health care professionals, such as nurses, clinical research coordinators, and health care experts who have received appropriate training and have the necessary expertise to conduct data collection and assessments in a clinical research setting. Their training ensures adherence to research protocols and ethical guidelines, guaranteeing accurate and consistent data gathering during clinical appointments. Two months after hospital discharge, we will mail all patients a letter offering a follow-up consultation in the outpatient clinic. Patients who agree to attend will be offered 2 appointments, consisting of an anamnesis and a physical examination, and given several self-administered tests in the waiting room. We will allocate 15 minutes for the questionnaire completion in the waiting room. In the first visit (T1, 3 months), the following data will be additionally collected: demographic characteristics like age, sex, and BMI (kg/m^2^); each participant’s medical history, such as the date of the first positive COVID-19 test (PCR or antigen) and smoking, alcohol, and drug consumption status; comorbidities and usual medication intake; and screening of symptoms and severity of the acute disease. Data collection in the second visit (T2, 6 months) will include post–COVID-19 symptoms, the Chalder Fatigue Scale [26] (CFQ-11), the Hospital Anxiety and Depression Scale (HADS) [27], the 14-item Posttraumatic Stress Scale (PTSS-14) adapted to COVID-19, and the EQ-5D [28] questionnaire.

### Ethical Considerations

This study involves human participants and has adhered to all applicable ethical standards and procedures. The Algarve University Hospital Centre Ethical Committee approved this study (141/21; Multimedia Appendix 2). The patients’ race will not be included in the study because the ethics committee did not approve this differentiation. A paper-based consent form was previously approved by the ethics committee. This consent form has 2 components: information for the participant (regarding the project) and declaration of informed consent (to date and sign, in case of acceptance). The informed consent procedure, mailed to potential participants, will provide them with detailed information about the study’s objectives, risks and benefits of participation, and data management practices (Multimedia Appendix 3). The study will follow ethical guidelines and regulations related to informed consent, and participants will have the opportunity to ask questions and provide voluntary consent before enrolling. Participants will be given a month for reflection before written informed consent. As for data management, the study will implement a secure and confidential system that uses data encryption and secure storage procedures to protect participant confidentiality and comply with data protection laws and regulations. Only authorized individuals who have a legitimate need to access the data will be permitted to do so, and any data sharing will be done in compliance with the relevant ethical and legal requirements. As applicable, all procedures from this investigation followed the Declaration of Helsinki. All researchers will comply with the Data Protection Acts of their respective academic institutions.

### Primary Outcome

#### Fatigue

A validated fatigue assessment tool will be used at the follow-up visit to capture a broader range of participants. This will provide a more comprehensive understanding of the relationship between fatigue and mental symptoms in individuals with long COVID. Fatigue will be measured using the CFQ-11 [26]. This is an 11-item self-report measure of physical and mental fatigue. Participants rate their fatigue experienced over the past month compared to their usual energy levels using a 4-point Likert scale ranging from 0 (“less than usual”) to 3 (“much more than usual”) [29]. Scores range from 0 to 33. A bimodal version can also be calculated, where scores range from 0 to 11, with a cutoff score of 4 or more indicating a case of fatigue [29]. Reliability coefficients for the CFQ-11 have been shown to range between 0.8 and 0.9 [29-31] in both patients with chronic fatigue and the general population. This study will use only the Likert scale to reduce the risk of ceiling effect bias.

### Secondary Outcomes

#### Depression and Anxiety

Symptoms of depression and anxiety will be assessed using the HADS [27], a practical, validated tool for assessing symptom severity in anxiety and somatic disorders in psychiatric and primary care settings and the general population [32]. This measure comprises 14 items, 7 measuring symptoms of anxiety and 7 measuring symptoms of depression. The scores are categorized into different ranges: 0 to 7 as “normal,” 8 to 10 as “mild,” 11 to 14 as “moderate,” and 15 to 21 as “severe.” The HADS questionnaire has been validated in many different languages and settings [32-34].

#### HRQoL Assessment

HRQoL will be assessed using the original EQ-5D questionnaire, which comprises 2 parts. The first part is the EQ-5D-3L questionnaire [28], a health state classification scheme of 5 items (mobility, self-care, usual activities, pain/discomfort, and anxiety/depression), each having 3 alternatives (1=no problems, 2=moderate problems, and 3=severe problems). The combination of answers for the 5 items represents the health state (given as an index), ranging from 0 (worst possible health state) to 1 (best possible health state). The second part comprises the EuroQol Visual Analog Scale (EQ-VAS), which represents health states in a range from 0 (the worst possible health state) to 100 (the best possible health state). The EQ-5D can distinguish between the health conditions of patients with diverse injuries [35,36] and has been validated in several populations [37].

#### PTSD Assessment

This study includes an assessment of PTSD as a secondary measure. It aims to evaluate the potential impact of PTSD on the primary outcome of fatigue and provide a better characterization of the study population. Although previous PTSD to COVID-19 infection is an exclusion criterion, some participants may still have experienced PTSD symptoms after their COVID-19 infection, so evaluating the prevalence and severity of these symptoms in the study population could be relevant.

Given that the “gold standard” clinician-administered, semistructured psychiatric interviews to diagnose PTSD symptoms were not feasible during the workload of the COVID-19 follow-up clinic, we chose the patient-reported outcome instrument PTSS-14. This scale was developed based on the Diagnostic and Statistical Manual of Mental Disorders, Third Edition, Revised (DSM-III-R) criteria for PTSD and subsequently validated for patients following intensive care [38]. Patients were asked to assess the frequency of 10 common PTSD symptoms on a 7-point Likert scale from 1 (never) to 7 (always). Item scores were summed up to a total score (ranging from 10 to 70). A total score above 35 was suggested to indicate clinically relevant PTSD symptoms. A similar 14-item version of PTSS was later developed to reflect the changes in the fourth edition of the DSM (DSM-IV), where a cutoff value of 45 indicated presumable PTSD [39]. We modified the latter to include COVID-19 patients—whenever the term “intensive care unit (ICU)” appeared, we substituted it with “COVID-19.” We applied the same diagnostic cutoff to this adapted scale.

### Association of Mental Illness Symptoms With the Severity of Acute COVID-19 

COVID-19 severity will be categorized according to the WHO classification [40]. The classification includes the following: mild illness (mild symptoms without the radiographic appearance of pneumonia); pneumonia (having symptoms and the radiographic evidence of pneumonia, with no requirement for supplemental oxygen); severe pneumonia (having pneumonia, including one of the following: respiratory rate >30 breaths/minute, severe respiratory distress, or blood oxygen saturation level ≤93% while at rest and breathing room air); and critical cases (eg, respiratory failure requiring invasive mechanical ventilation or nasal high flow oxygen, septic shock, other organ failure occurrence, or admission to the ICU).

### Statistical Analyses

The statistical analysis methodology is designed to align with the study’s defined objectives. All analyses will be conducted using SPSS (version 27; IBM Corp) and R (R Foundation for Statistical Computing) software. The analyses will be carried out in 5 phases, as outlined in Textbox 2.

**The 5 phases of the statistical analyses carried out in this study.**

- Phase 1: Characterization of the patient sample
  1.1. Sociodemographic analysis of the variables collected at T1 and the variables from the surveyed instruments at T2. This involves descriptive analysis, both univariate and bivariate, which will be differentiated by:
    1.1.1. Quantitative variables: mean and SD, coefficient of variation, and median and IQR
    1.1.2. Qualitative variables (nominal and ordinal categorical): analysis of distributions through absolute and relative frequencies, including absolute and relative accumulated frequencies
    1.1.3. Quantitative variables will be reported alongside appropriate tests for normal distribution to ensure the accuracy and validity of our statistical analyses.
  1.2. Bivariate and descriptive analysis using 2D tables will be calculated for all qualitative variables belonging to T1 with all instrument scores collected at T2: Chalder Fatigue Scale (CFQ-11), Hospital Anxiety and Depression Scale (HADS), 14-item Posttraumatic Stress Scale (PTSS-14) adapted to COVID -19, and EQ- 5D plus recorded COVID-19 symptoms. The previous points will be reiterated in this bivariate context, utilizing the computational aid of split->file categorical variables in situations where one variable is metric and the other is categorical.
  1.3. Internal consistency and reliability analysis of the T2 instruments: calculation of the Cronbach alpha (general and partial) for all ordinal items and item subgroups differentiated by the subscales defined by the respective authors
- Phase 2: Determination of fatigue incidence caused by long COVID and its relationship with T1 and T2 variables
  2.1. Procedures for determining fatigue cutoff points in this cohort of patients:
    2.1.1. Bivariate analysis using optimal binning procedures for categorical variables (age class, sex, and BMI) with the total fatigue score, as well as partial scores of physical and psychological fatigue
    2.1.2. Multivariate analysis by constructing models in the format of decision trees via the CHAID (chi-square automatic interaction detection) algorithm [41], with total and partial fatigue scores as dependent variables
  2.2. Inferential analysis based on variables resulting from 2.1.1 and/or 2.1.2, considering the complexity of variables in T1 and T2:
    2.2.1. (1) Chi-Square tests to capture the association of categorical variables; (2) Shapiro-Wilk tests to verify the distribution of quantitative variables; (3) tests for comparing 2 or more population means: parametric analysis of variance (ANOVA) test and nonparametric (Kruskal-Wallis) test; and (4) correlation tests (Spearman or Pearson) to verify the degree of association between T2 instrument scales
- Phase 3: Determination of patient groups based on fatigue differentiation
  3.1. Conducting classification analysis using clustering techniques, specifically the 2-step cluster analysis, to explore variables with greater power in differentiating groups of patients
- Phase 4: Exploration of the best combination of T1 and T2 variables capable of discriminating patient fatigue levels through a discriminant multivariate analysis
- Phase 5: Investigation of the association between phase 3 groups and all T2 instrument scales using chi-square association tests

### Power and Sample Size

We plan to conduct a prospective multicentric observational study to investigate the prevalence and severity of mental illness symptoms and fatigue in individuals with long COVID. Based on previous studies and expert opinion, we expect the prevalence of fatigue and mental symptoms in this population to be around 50% [42,43]. We aim to detect a minimum effect size of 0.2 with a power of 80% and a significance level of *P=.*05.

Using these assumptions, we performed a sample size power analysis using a 2-sample *t* test. We found that a total sample size of 200 participants (100 with adverse mental health symptoms and 100 without) would be required to detect the minimum effect size of 0.2 with 80% power and a significance level of *P=*.05. The required sample size was calculated using an a priori power analysis with an online calculator [44]. To compensate for a projected 25% loss to follow-up, we aimed for 250 participants.

We plan to recruit participants from multiple hospitals and primary care centers across Portugal, focusing on individuals who have been diagnosed with long COVID and are experiencing mental illness symptoms and/or fatigue. Recruitment will be closely monitored throughout the study to assess the pace at which participants are enrolled. The need for sample size adjustment will be determined based on the observed recruitment rates and the accumulating data. We plan to conduct an interim analysis after the recruitment of the first 100 patients.

## Results

**Figure 2:**
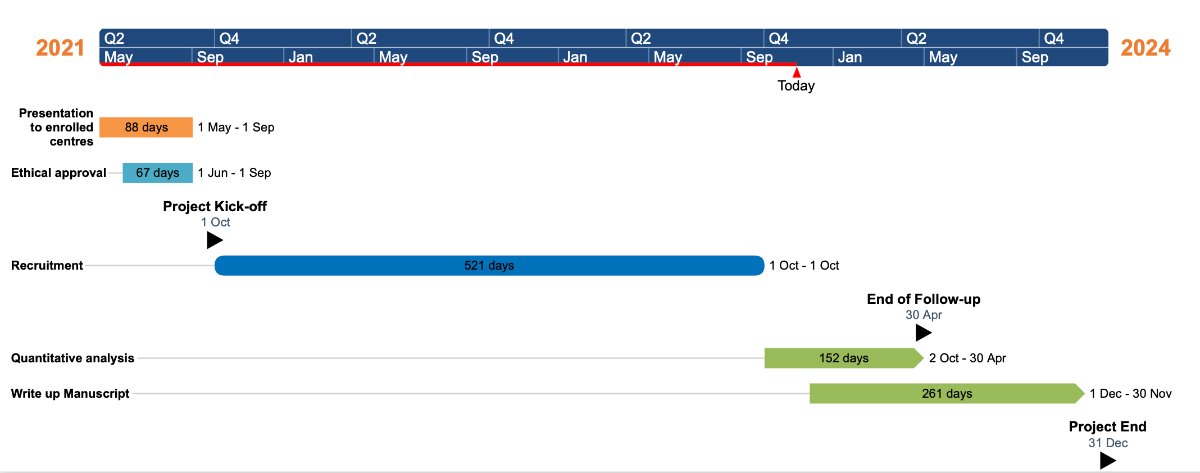
Study timeline.

This study received no specific grant from any funding agency in public, commercial, or not-for-profit sectors. Recruitment began in October 2021 at Portimão Hospital and Alvor Hospital, in June 2022 at Faro Hospital and São Sebastião Hospital, and in October 2022 at Fernando Fonseca Hospital. The deadline for the end of the recruitment period in all centers was December 2023. The preliminary study results will be published in a peer-reviewed international medical journal after October 2023. The study timeline is shown in Figure 2. This study is included as part of the first author’s (LP) PhD thesis. The initial study results were presented at the PhD interview in November 2023. We aim to publish the study in an indexed scientific journal and present the results at national and international congresses.

We plan to enroll 250 participants, including 100 with adverse mental health symptoms and 100 without, to explore the intricate relationship between adverse mental health symptoms and both physical and psychological fatigue in individuals recovering from long COVID. Preliminary analyses indicate a compelling connection, wherein patients who report heightened levels of depression and anxiety also tend to experience increased fatigue. These findings underscore the importance of considering mental health as a pivotal factor in understanding the enduring impact of COVID-19 beyond the acute phase. In addition to depression and anxiety, our study explores the persistence of PTSD symptoms in a subset of participants. Initial results suggest that, even after recovery from the acute phase of the infection, a proportion of individuals continue to grapple with the psychological repercussions of the virus. This observation aligns with emerging evidence suggesting a prolonged psychological impact of COVID-19, emphasizing the need for comprehensive mental health support for patients after contracting COVID-19. The identified links between mental health symptoms and fatigue have broad implications for the holistic care of this patient population. Understanding these connections can guide targeted interventions, emphasizing the importance of addressing mental well-being alongside physical recovery. As we delve deeper into the data and conduct further analyses, a more nuanced understanding of these relationships will emerge, informing future health care strategies and interventions.

## Discussion

### Expected Findings

As the COVID-19 pandemic ceases, more patients enter the chronic phase of the disease. Identifying groups at high risk of cognitive and psychiatric dysfunction may allow for targeted intervention to effectively meet their physical, neurological, and psychological health care needs. Our study, conducted in the aftermath of the COVID-19 pandemic, provides a unique opportunity to investigate the persisting health effects of the virus on those who have recovered from the acute phase of the disease. We specifically aim to explore post–COVID-19 fatigue and mental health disorders using specific tools to separate physical and psychological fatigue symptoms. We also use a prospective cohort multicenter study design, which is less prone to bias, and we aim to include both hospitalized and nonhospitalized patients, covering all degrees of acute COVID-19 severity. We anticipate that our research will reveal a significant association between the presence and severity of mental health symptoms, including depression, anxiety, and PTSD, and the degree of physical and psychological fatigue in patients following COVID-19. We expect to observe that patients experiencing more pronounced mental symptoms will report higher levels of physical and psychological fatigue.

The potential links between mental symptoms and fatigue suggest a connection between the mental well-being of patients post COVID-19 and their experience of fatigue, encompassing both physical and psychological dimensions. The existing research has indicated a high prevalence of anxiety and depression symptoms among patients hospitalized with COVID-19 infection, with a quarter of patients experiencing at least mild symptoms of acute stress disorder [42]. Moreover, a study conducted in Iran after the outbreak of COVID-19 found a correlation between the prevalence of chronic fatigue syndrome and PTSD [45]. The study reported that 5.8% of subjects experienced PTSD symptoms 6 months after the onset of SARS-CoV-2 infection. Interestingly, the study also noted that female sex was associated with a higher risk of fatigue. At the same time, variables such as oxygen saturation at admission, primary symptoms, ICU admission, and laboratory test parameters did not show a significant association with fatigue occurrence.

Based on these findings, we anticipate that patients post COVID-19 may exhibit more pronounced symptoms of depression and anxiety and heightened levels of physical and psychological fatigue. This insight emphasizes the importance of considering mental health as a crucial factor in understanding the long-term effects of COVID-19. It underscores that the impact of the virus extends beyond the acute illness phase and can manifest in persistent mental symptoms that influence patients’ overall well-being. Moreover, it can imply that addressing anxiety symptoms in post–COVID-19 care may have a positive impact on reducing fatigue and improving patients’ quality of life. We also aim to explore the prevalence and severity of PTSD symptoms in a subset of patients following COVID-19. While this is not the primary focus of our research, assessing the presence of PTSD symptoms in this study population allows for a better characterization of the patient group. Some participants continue to experience PTSD symptoms following COVID-19 infection, suggesting that the psychological impact of the virus can be long-lasting. These findings may shed light on the need for comprehensive mental health support for patients post COVID-19.

The implications of our potential findings are widespread. They underscore the importance of integrated care for patients post COVID-19 that addresses both physical and mental health aspects. Clinicians and health care providers will then be aware of the potential for persistent mental symptoms and their impact on patients’ fatigue levels and overall HRQoL.

### Limitations

Our study’s limitations include the possibility of sampling bias, as individuals with more severe symptoms of fatigue or other mental health symptoms may be less likely to participate. This possibility could result in underrepresentation and bias within the study’s findings. To minimize this, we will use diverse recruitment methods across multiple sites and collect data on participants’ reasons for declining participation. We will also provide detailed information about the study and its potential benefits to participants while ensuring an ethical and respectful recruitment process sensitive to participants’ health concerns and symptoms.

We also acknowledge the limitations related to our exclusion criteria and use of the CFQ-11, which has yet to undergo specific validation for patients with COVID-19. This recognition underscores the importance of future research endeavors to validate the applicability of the CFQ-11 within this patient population, despite its utilization in prior studies [46-48].

While there are valid concerns regarding the exclusion criteria and assessment of psychiatric disorders in our study—including the fact that assessing preexisting fatigue may be difficult—we have devised a plan to mitigate these concerns. We intend to meticulously collect detailed medical histories and review the medical records of all enrolled patients to ensure the precise application of the exclusion criteria. However, a notable limitation of our study arises from the absence of a baseline assessment conducted before the onset of COVID-19 infection. This absence impedes our ability to comprehensively evaluate the influence of COVID-19 on various health parameters, such as fatigue and mental health. The absence of a baseline assessment may also limit our capacity to distinguish between preexisting conditions and post–COVID-19 effects, which is a key consideration in understanding the full spectrum of the virus’s impact on individuals. Additionally, while we acknowledge that the measurements used in the study are based on self-report—and therefore subjective—we emphasize that self-reported questionnaires are commonly used in scientific studies. These questionnaires hold significance in assessing the extent of physical and psychological fatigue in patients following COVID-19. Despite these limitations, our study is poised to make a significant contribution to the existing literature by shedding light on the impact of COVID-19 on mental health. This research has the potential to guide future treatment and management strategies, serving as a valuable resource for the health care community.

### Conclusion

This study protocol outlines a critical investigation into the lingering physical and psychological effects of long COVID. It emphasizes the importance of understanding the ongoing health challenges individuals face even after recovering from the acute phase of the virus. This study’s research questions focus on assessing the potential correlations of mental illness symptoms (ie, physical and psychological fatigue) among patients post COVID-19.

In the aftermath of a global pandemic, this research is timely and crucial. It has the potential to inform health care strategies and interventions that provide targeted and holistic care to individuals grappling with long COVID. Ultimately, this study aims to improve patient outcomes, enhance the quality of life for those affected, and contribute to broader efforts to address the multifaceted health implications of this unprecedented global crisis.

## Appendix

Multimedia Appendix 1 Study clinical research form (CRF).

Multimedia Appendix 2 Ethical approval.

Multimedia Appendix 3 Informed consent form.

Multimedia Appendix 4 SPIRIT checklist.

## Abbreviations

CFQ-11: Chalder Fatigue Scale
CRF: clinical research form
DSM-III-R: Diagnostic and Statistical Manual of Mental Disorders, Third Edition, Revised
DSM-IV: Diagnostic and Statistical Manual of Mental Disorders, Fourth Edition
EQ-VAS: EuroQol Visual Analog Scale
HADS: Hospital Anxiety and Depression Scale
HRQoL: health-related quality of life
ICU: intensive care unit
MERS: Middle East respiratory syndrome
PTSD: posttraumatic stress disorder
PTSS-14: 14-item Posttraumatic Stress Scale
RT-PCR: reverse-transcription polymerase chain reaction
SARS: severe acute respiratory syndrome
SPIRIT: Standard Protocol Items: Recommendations for Interventional Trials
WHO: World Health Organization
