# Supplementary material for: Fatigue and Mental Illness Symptoms in Long COVID: Protocol for a Prospective Cohort Multicenter Observational Study
Source: JMIR Res Protoc. 2024 Jan 19;13:e51820. doi: 10.2196/51820 (PMC10837758; doi:10.2196/51820)
Supplement: Multimedia Appendix 1 [file resprot_v13i1e51820_app1.pdf]

HOSPITAL ___________________________ DOCTOR NAME _______________

A. INCLUSION DATA

1. Patient code: ____________________
   (Assigned randomly and only to the knowledge of each principal investigator by Center)
2. Date of positive PCR/TRAG:____/_____/_________
3. Origin of referral for the post-COVID medical consultation:
   Primary care? ___ Hospital? ___ Occupational medicine for health care workers? ___

B. SOCIODEMOGRAPHIC DATA

1. Birth date: ____/_____/_________
2. Sex (Female/Male)
3. Weight: ____ Kg Heigt: __,___ m BMI: _______
4. Schooling: ________________________ Profession: ___________________________
5. Did you lose your job during de pandemia? Yes/No
6. Household: ______________________cohabitants

C. BACKGROUND

1. Smoking habits (Never Smoker / Ex-Smoker_____ UPY / Smoker______UPY)
2. Alcohol comsumption_________________ Drugs Comsumption__________________
3. Comorbidities(Yes/No):
   i. Arterial hypertension
   ii. Diabetes
   iii. Heart disease (Which?____)
   iv. Asthma
   v. COPD
   vi. Immunossupression (Which?____)
   vii. Psychiatric disease, diagnose by psychiatrist
   (Which? ____)
   viii. Neurological disease
   (Which?____)
4. Medication
   i. Previous to COVID-19 ____
   ii. After COVID-19: Corticoids (Yes/No), name/dose? _____________________________
   Other medications ____

T1 – LONG COVID (Persistent symptoms 3 months post-COVID-19)

A) SEVERITY OF ACUTE DISEASE

1. Mild disease: mild symptoms with no evidence of pneumonia or hypoxemia.
2. Moderate disease**:** pneumonia (fever, cough, dyspnea, tachypnea) but with peripheral O2 saturation ≥ 90% in room air, and without hemodynamic instability.
3. Severe disease**:** pneumonia and at least one of the following criteria: tachypnea >30 cycles per minute; dyspnoea; pulse oximetry O2 < 90% in ambient air; hemodynamic instability.

Hospital Admission? Y/N   Intensive Care? Y/N   Invasive Mechanical Ventilation? Y/N

B) SYMPTOMS

Cough ___ Fatigue ___ Difficulty breathing ___ Chest pain ___

Muscle tension and pain ____ (Where? ____)

Pain in the joints ____ (Were? ____)

Headaches ___ Fever ___ Loss of appetite ___ Loss of taste ___ Loss of smell ___

Conjunctivitis ___ Misty vision ___ Decreased visual acuity ___ Memory loss ___ Brain fog ___

Daytime hypersomnolence ___ Insomnia ___Anxiety ___ Depression ___ Aphasia ___ Hemiparesis ___

Skin lesions ___ (Which and where? ____)

Diarrhea ___ Vomiting ____ Other ____

C) MEDICATION DURING HOSPITALIZATION? Y / N

Metilprednisolone (Total dose_____mg)   Dexametasone (Total dose_____mg)

Remdesivir (Total dose______mg)   Tocilizumab (Total dose ______mg) Others ___________________

D) SOCIAL ISOLATION

Number of days you have been isolated at home _______ Days (Alone or with family members?)

Number of days in hospital sanitary isolation_______ Days

Pós – COVID-19

t1 (8 months after PCR/TRAG positive test): ____/_____/_________

A) SYMPTOMS

Cough ___ Fatigue ___ Difficulty breathing ___ Chest pain ___

Muscle tension and pain ____ (Where? ____)

Pain in the joints ____ (Were? ____)

Headaches ___ Fever ___ Loss of appetite ___ Loss of taste ___ Loss of smell ___

Conjunctivitis ___ Misty vision ___ Decreased visual acuity ___ Memory loss ___ Brain fog ___

Daytime hypersomnolence ___ Insomnia ___Anxiety ___ Depression ___ Aphasia ___ Hemiparesis ___

Skin lesions ___ (Which and where? ____)

Diarrhea ___ Vomiting ____ Other ____

B) Fatigue Chalder Scale scoring

Depression subscale ____ points

Anxiety subscale ____ points

Total score ____ points

C) HADS scoring

Depression subscale ____ points

Anxiety subscale ____ points

D) PTSS 14 scoring

Total score ____ points

E) EQ-5D scoring

Health status EQ-VAS____ % EQ-5D-3L (attach only)

chalder fatigue scale

name: ______________________________ date: _______

We would like to know more about any problems you have had with feeling tired, weak or lacking in energy in the last month. Please answer ALL the questions by ticking the answer which applies to you most closely. If you have been feeling tired for a long while, then compare yourself to how you felt when you were last well. Please tick only one box per line.

	less than usual	***no more than usual***	***more than usual***	***much more than usual***
do you have problems with tiredness?				
do you need to rest more?				
do you feel sleepy or drowsy?				
do you have problems starting things?				
do you lack energy?				
do you have less strength in your muscles?				
do you feel weak?				
do you have difficulties concentrating?				
do you make slips of the tongue when speaking?				
do you find it more difficult to find the right word?				
	better than usual	***no worse than usual***	***worse than usual***	***much worse than usual***
how is your memory?				

This scale can be scored "bimodally" with columns representing 0, 0, 1 & 1 and a range from 0 to 11 with a total of 4 or more qualifying for "caseness". Alternatively it can be scored in "Likert" style 0, 1, 2 & 3 with a range from 0 to 33. Mean "bimodal" score for CFS sufferers was 9.14 (SD 2.73) and for a community sample 3.27 (SD 3.21). Mean "Likert" score was 24.4 (SD 5.8) and 14.2 (SD 4.6).

total (0-33) =

Cella, M. and T. Chalder (2010). *"Measuring fatigue in clinical and community settings."* J Psychosom Res 69(1): 17-22. This study involved 361 CFS sufferers and 1615 individuals from the community. Average age was in the 30's. Fatigue levels were similar for males and females. A score of 29 discriminated between CFS sufferers and the community sample in 96% of cases and a score in the 30's discriminated in 100% of cases. The CFS sufferers also scored a mean of 26.99 on the Work & Social Adjustment Scale (W&SAS) with a SD of 8.6 (i.e. about 70% scoring between 18.4 and 35.6).

HOSPITAL ANXIETY AND DEPRESSION SCALE (HADS)

STUDY CODE ______________________________________Date: ___ / ___ / ___

Tick the box beside the reply that is closest to how you have been feeling in the past week. Don’t take too long over you replies: your immediate is best..

A1. **I feel tense or 'wound up':**

3 ( ) Most of the time

2 ( ) A lot of the time

1 ( ) From time to time, occasionally

0 ( ) Not at all

D2. **I still enjoy the things I used to enjoy:**

0 ( ) Definitely as much

1 ( ) Not quite so much

2 ( ) Only a little

3 ( ) Hardly at all

A3. **I get a sort of frightened feeling as if something awful is about to happen:**

3 ( ) Very definitely and quite badly

2 ( ) Yes, but not too badly

1 ( ) A little, but it doesn't worry me

0 ( ) Not at all

D4. **I can laugh and see the funny side of things:**

0 ( ) As much as I always could

1 ( ) Not quite so much now

2 ( ) Definitely not so much now

3 ( ) Not at all

A5. **Worrying thoughts go through my mind:**

3 ( ) A great deal of the time

2 ( ) A lot of the time

1 ( ) From time to time, but not too often

0 ( ) Only occasionally

D6. **I feel cheerful:**

0 ( ) Not at all

1 ( ) Not often

2 ( ) Sometimes

3 ( ) Most of the time

A7. **I can sit at ease and feel relaxed:**

0 ( ) Definitely

1 ( ) Usually

2 ( ) Not Often

3 ( ) Not at all

D8. **I feel as if I am slowed down:**

3 ( ) Nearly all the time

2 ( ) Very often

1 ( ) Sometimes

0 ( ) Not at all

A9. **I get a sort of frightened feeling like 'butterflies' in the stomach:**

0 ( ) Not at all

1 ( ) Occasionally

2 ( ) Quite Often

3 ( ) Very Often

D10. **I have lost interest in my appearance:**

3 ( ) Definitely

2 ( ) I don't take as much care as I should

1 ( ) I may not take quite as much care

0 ( ) I take just as much care as ever

A11. **I feel restless as I have to be on the move:**

3 ( ) Very much indeed

2 ( ) Quite a lot

1 ( ) Not very much

0 ( ) Not at all

D12. **I look forward with enjoyment to things:**

0 ( ) As much as I ever did

1 ( ) Rather less than I used to

2 ( ) Definitely less than I used to

3 ( ) Hardly at all

A13. **I get sudden feelings of panic:**

3 ( ) Very often indeed

2 ( ) Quite often

1 ( ) Not very often

0 ( ) Not at all

D14. **I can enjoy a good book or radio or TV program:**

0 ( ) Often

1 ( ) Sometimes

2 ( ) Not often

3 ( ) Very seldom

*Please check you have answered all the questions

Scoring:

Total score: Depression (D) ___________ Anxiety (A) ______________

0-7 = Normal
8-10 = Borderline abnormal (borderline case)
11-21 = Abnormal (case)

Zigmond, AS; Snaith, RP (1983). "The hospital anxiety and depression scale". Acta Psychiatrica Scandinavica. 67 (6): 361–370.

Pais-Ribeiro J, Silva I, Ferreira T, Martins A, Meneses R, Baltar M. Validation study of a Portuguese version of the Hospital Anxiety and Depression Scale. Psychol Health Med. 2007 Mar;12(2):225-35;

EQ-5D-3 Questionnaire

Health Questionnaire (EQ-5D-5L)

Under each heading, please tick the ONE box that best describes your health TODAY.

MOBILITY

☐$_1$ I have no problems in walking about
☐$_2$ I have slight problems in walking about
☐$_3$ I have moderate problems in walking about
☐$_4$ I have severe problems in walking about
☐$_5$ I am unable to walk about

SELF-CARE

☐$_1$ I have no problems washing or dressing myself
☐$_2$ I have slight problems washing or dressing myself
☐$_3$ I have moderate problems washing or dressing myself
☐$_4$ I have severe problems washing or dressing myself
☐$_5$ I am unable to wash or dress myself

USUAL ACTIVITIES *(e.g. work, study, housework, family or leisure activities)*

☐$_1$ I have no problems doing my usual activities
☐$_2$ I have slight problems doing my usual activities
☐$_3$ I have moderate problems doing my usual activities
☐$_4$ I have severe problems doing my usual activities
☐$_5$ I am unable to do my usual activities

PAIN / DISCOMFORT

☐$_1$ I have no pain or discomfort
☐$_2$ I have slight pain or discomfort
☐$_3$ I have moderate pain or discomfort
☐$_4$ I have severe pain or discomfort
☐$_5$ I have extreme pain or discomfort

ANXIETY / DEPRESSION

☐$_1$ I am not anxious or depressed
☐$_2$ I am slightly anxious or depressed
☐$_3$ I am moderately anxious or depressed
☐$_4$ I am severely anxious or depressed
☐$_5$ I am extremely anxious or depressed

UK (English) © 2009 EuroQol Group EQ-5D™ is a trade mark of the EuroQol Group

EQ-VAS Questionnaire

Health Questionnaire (EQ-5D-5L)

- We would like to know how good or bad your health is **TODAY**.
- This scale is numbered from 0 to 100.
- 100 means the <u>best</u> health you can imagine.
  0 means the <u>worst</u> health you can imagine.
- Mark an X on the scale to indicate how your health is **TODAY**
- Now, please write the number you marked on the scale in the below.

YOUR HEALTH TODAY = [ ]

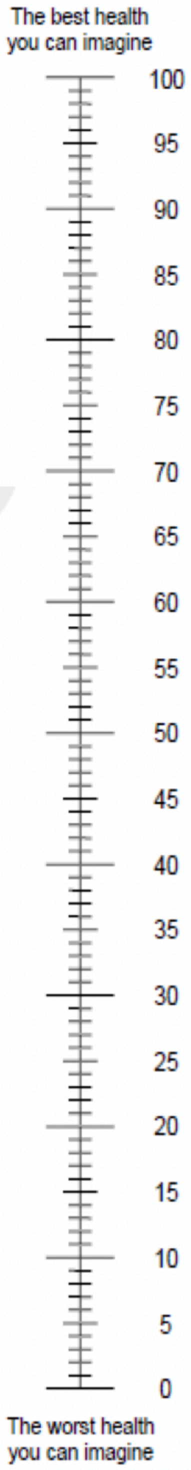

UK (English) © 2009 EuroQol Group EQ-5D™ is a trade mark of the EuroQol Group
