# Supplementary material for: Fatigue and Mental Illness Symptoms in Long COVID: Protocol for a Prospective Cohort Multicenter Observational Study
Source: JMIR Res Protoc. 2024 Jan 19;13:e51820. doi: 10.2196/51820 (PMC10837758; doi:10.2196/51820)
Supplement: Multimedia Appendix 2 [file resprot_v13i1e51820_app2.pdf]

Comissão de Ética para a Saúde

tel. 41110 / 8596
e-mail comissão.etica@chalgarve.min-saude.pt

Exm.(a) Sr.(a)
Conselho de Administração
Centro Hospitalar Universitário do Algarve

s. ref. s. com. n. ref./data 141/21, 06.09.2021

Assunto: Estudo" Sequelas neuropsicológicas como fator de risco para fadiga pós - COVID 19"

De acordo com o emitido pelo Presidente da CES de dia 21-07-2021 o questionário exclui qualquer dado de identificação do paciente. O parecer é positivo.

A Comissão de Ética para a Saúde,
