# Supplementary material for: Fatigue and Mental Illness Symptoms in Long COVID: Protocol for a Prospective Cohort Multicenter Observational Study
Source: JMIR Res Protoc. 2024 Jan 19;13:e51820. doi: 10.2196/51820 (PMC10837758; doi:10.2196/51820)
Supplement: Multimedia Appendix 3 [file resprot_v13i1e51820_app3.pdf]

Consentimento Informado para Projeto de Investigação

“Sequelas neuropsicológicas como fator de risco para fadiga pós-COVID-19”

Este consentimento informado refere-se a um projecto de investigação sobre as consequências da infecção por SARS-CoV-2 (doença COVID-19) a nível psicológico, no qual foi convidado a participar.

Nome do Investigador Principal: Dra. Lígia Pires
Nome da Organização: Centro Hospitalar Universitário do Algarve (CHUA)

Este formulário de consentimento informado tem 2 componentes:

- Informação para o participante (informação sobre o projeto)
- Declaração de consentimento do participante (para preencher por escrito, caso aceite participar)

Caso aceite participar, ser-lhe-á fornecida uma cópia do consentimento informado, após o seu preenchimento por escrito.

Informação para o participante

Introdução

A COVID-19 é uma doença de aparecimento recente, sendo o conhecimento científico sobre a mesma ainda limitado. Na literatura tem sido relatada a presença de sintomas de fadiga até aos 6-7 meses após a doença aguda por COVID 19, semelhantes à síndrome da fadiga crónica, observada também após outras infeções virais. A síndrome da fadiga crónica pode ter relação com as alterações neuropsicológicas pós-COVID-19, pelo que dentificar as sequelas pós-COVID-19 para além das sequelas respiratórias, é fundamental para o desenvolvimento de métodos de prevenção e tratamento precoces.

Este projeto integra todos os doentes que tiveram COVID 19 HÁ PELO MENOS 6 MESES.
Na sequência do convite que lhe foi feito para participar neste estudo, ser-lhe-á fornecida informação sobre o mesmo.
Caso tenha dúvidas pode pedir informação adicional.

Objetivo da investigação

Pretende-se avaliar se existem sequelas neuropsicológicas e/ou fadiga após COVID-19, e qual o seu impacto na qualidade de vida.

Seleção de participantes

São convidados a participar todos os pacientes adultos (≥ 18 anos) que tenham sido diagnosticados com COVID-19, e que recorram á consulta pós-COVID-19 no Centro Hospitalar Universitário do Algarve (CHUA), nomeadamente nas Unidades de Faro e Portimão, no período compreendido entre 1 de julho de 2021 e 31 de janeiro de 2022.
Não serão incluídos no estudo doentes que previamente à COVID 19 já tinham doenças que provocavam fadiga (como fibromialgia), doenças neurológicas (AVC, Parkinson, Alzeimer) ou doenças psiquiátricas.

Participação voluntária

A participação neste estudo é voluntária, sendo o participante livre de recusar participar ou de desistir da sua participação em qualquer altura.
Caso não queira participar ou desistir da sua participação durante o estudo, será mantido o habitual seguimento clínico de acordo com o melhor conhecimento e julgamento do médico referenciador, sem prejuízo para o participante.
Este projeto de investigação foi aprovado pela Comissão de Ética do CHUA.

Procedimentos

O estudo decorrerá como parte do seguimento dos doentes que foram diagnosticados com COVID-19 e recorreram às consultas de seguimento pós-COVID-19.

Na consulta de seguimento será ainda feita uma avaliação clínica, solicitado o preenchimento de questionários com escalas psicológicas, do estado de saúde e de fadiga.

Duração
Este estudo tem a duração de uma consulta por participante.

Riscos
Trata-se de um estudo de observação, sem intervenção.

Benefícios
A participação neste estudo irá permitir aumentar o conhecimento sobre as possíveis consequências da doença COVID-19 a nível psicológico e consequentemente adequar de forma mais eficaz os tratamentos disponíveis. Os dados recolhidos serão disponibilizados ao seu médico hospitalar.

Reembolsos
Não serão fornecidos reembolsos pela participação.

Confidencialidade
A identidade dos participantes não será divulgada (apenas os médicos hospitalares terão conhecimento da participação). A confidencialidade será garantida anonimizando os dados recolhidos.

Divulgação de resultados
Os resultados deste estudo serão divulgados aos médicos hospitalares que fazem o seguimento dos participantes. Os conhecimentos adquiridos serão publicados em revistas científicas e apresentados em eventos científicos, de forma a difundir os dados obtidos respeitantes às sequelas da COVID-19.

Pessoa de contacto
Se tiver qualquer questão relativa ao estudo agora ou posteriormente, poderá contactar:
- Dra. Ligia Pires: ligia.vicente@chalgarve.min-saude.pt

Declaração de consentimento do participante

Declaro ter lido e compreendido este documento, bem como as informações verbais que me foram fornecidas. Foi-me garantida a possibilidade de, em qualquer altura, recusar participar neste estudo sem ter de dar qualquer tipo de justificação e sem que a prestação de cuidados necessários ao meu estado de saúde seja afectada. Desta forma aceito participar neste estudo.

Nome do participante (legível) ____

Assinatura do participante ____

Data (dia/mês/ano) ____

Declaração do investigador/pessoa a recolher o consentimento

Li de forma atenta a folha de informação ao potencial candidato, e tanto quanto me é dado a entender, confirmo que o participante percebeu que:

1. A participação no presente estudo inclui avaliação clínica em consulta, preenchimento de questionários.
2. O participante dá o seu consentimento livre de participar, sendo que em qualquer altura poderá desistir.

Confirmo que o participante teve oportunidade de colocar questões sobre o estudo, e que todas as questões foram respondidas. Confirmo que o indivíduo não foi coagido a dar consentimento e que o consentimento foi dado livre e voluntariamente. Uma cópia do documento foi fornecida ao participante.

Nome da pessoa que recolheu o consentimento ____

Assinatura da pessoa que recolheu o consentimento ___________________________________

Data (dia/mês/ano) ____
